# 基于碳基磁性材料的磁固相萃取技术在食品分析应用中的研究进展

**DOI:** 10.3724/SP.J.1123.2020.05038

**Published:** 2021-04-08

**Authors:** Yingmin LIAO, Xiaojia HUANG, Zhuozhuo WANG, Rui GAN

**Affiliations:** 1.厦门大学嘉庚学院, 河口生态安全与环境健康福建省高校重点实验室, 福建 漳州 363105; 1. Estuarine Ecological Security and Environmental Health Key Laboratory of Provincial University,Tan Kah Kee College, Xiamen University, Zhangzhou 363105, China; 2.厦门大学环境与生态学院, 福建 厦门 361005; 2. College of Environment and Ecology, Xiamen University, Xiamen 361005, China

**Keywords:** 磁固相萃取, 碳基磁性材料, 吸附剂, 食品分析, 综述, magnetic solid phase extraction (MSPE), carbon based magnetic material, adsorbent, food analysis, review

## Abstract

食品中残留的痕量有毒物质严重威胁人体健康,对其进行分析十分必要。然而,食品中有毒物质种类多、量少、基质复杂,需选择适当的样品预处理技术进行提取和净化。磁固相萃取(MSPE)因具有操作简单、省时快速、无需离心过滤、环境友好等优点,被认为是一种高效的样品预处理技术并应用于食品分析中。MSPE中使用的磁性吸附剂的吸附容量和选择性是影响MSPE萃取效率和选择性高低的关键,对所建立分析方法的准确度起着关键作用。碳基磁性材料是具有价格低廉、来源丰富、比表面积大、化学稳定性好、吸附容量高、绿色环保等优点的一类新型功能性磁性材料,可以富集不同性质的有机、无机分析物,在环境分析、生物检测、污染治理等多个领域取得了较大进展。近年来,基于碳基磁性材料的MSPE技术在食品分析预处理领域逐渐得到应用,但尚处于起步阶段,存在巨大的应用潜力。该文以碳基类别(碳纳米管、石墨烯、金属有机骨架衍生碳、活性炭等)为主线,综述了采用MSPE技术,以碳基磁性材料为吸附剂,对食品样品中酯类、真菌毒素、多环芳烃、抗生素、生物碱、酚类、维生素、抗菌药等物质进行萃取,进而采用液相色谱法等进行分析的应用实例,同时阐述了该技术存在的问题,并对其发展方向做出了展望。该综述将为基于碳基磁性材料的MSPE技术在食品分析中的广泛应用提供理论依据和技术支撑。

## Abstract (en)

Trace toxic substances in food pose a serious threat to human health, and need to be detected and analyzed to ensure food safety. However, there are many kinds of toxic substances in food, with small amounts and complex matrices, making it necessary to select an appropriate sample pretreatment technology for extraction and purification. There are some disadvantages to sample pretreatment methods such as solid phase extraction and liquid-liquid extraction, in terms of poor selectivity, significant influence of matrix interference, large sample requirement, long extraction time, use of a large amount of harmful organic solvents, and cumbersome and time-consuming operation. Magnetic solid phase extraction (MSPE) combines the advantages of magnetic separation and traditional SPE technology, avoids time-consuming column loading, and can extract the target analyte efficiently. Because of its advantages, in that it has simple operation, is time-saving and fast, requires no centrifugal filtration, and is environmentally friendly, it is considered an efficient sample pretreatment technology and applied in food analysis. The adsorption capacity and selectivity of the magnetic adsorbent used in MSPE are the key factors affecting the extraction efficiency and selectivity of MSPE, and play a key role in the accuracy of the established method. Carbon-based magnetic materials are a type of new functional magnetic materials prepared by the co-precipitation of carbon-based materials (carbon nanotubes, graphene, metal-organic framework-derived carbon, or activated carbon) and magnetic materials. In order to endow carbon-based magnetic materials with the advantages of both, carbon materials and magnetic materials, while also reflecting the advantages of high specific surface area, good stability, low cost, environmental friendliness, excellent physical and chemical properties, high porosity, and high adsorption capacity, proper functional modification is needed. Carbon-based magnetic materials modified by functionalization can efficiently enrich organic and inorganic analytes with different properties, and have seen significant progress in environmental analysis, biological detection, pollution control, and other fields. In recent years, MSPE technology based on carbon-based magnetic materials has been gradually applied in food analysis and pretreatment, but its use is still in infancy and holds immense application potential. Reference to more than 50 papers published in SCI and Chinese core journals over the past four years reveals that carbon-based materials include carbon nanotubes modified by functional groups, reagents, or materials; graphene, graphene oxide, and reduced graphene oxide; carbon derived from a gold organic framework; activated carbon biochar; and nanodiamond. The harmful substances in food samples include esters, mycotoxins, polycyclic aromatic hydrocarbons, antibiotics, alkaloids, phenols, vitamins, and antibiotics. Based on the classification of carbon-based materials, this review reveals that carbon-based magnetic materials have good preconcentration ability for harmful substances in food samples. MSPE can be combined with GC-MS, liquid chromatography-high resolution mass spectrometry (LC-HRMS), ultra-fast liquid chromatography-tandem mass spectrometry (UFLC-MS/MS), ultra high performance liquid chromatography-Q-Exactive high resolution mass spectrometry (UHPLC-Q-Exactive HRMS), high performance liquid chromatography-diode array detection (HPLC-DAD), gas chromatography micro-electron capture detection (GC-μECD), high performance liquid chromatography fluorescence with post-column photochemical derivatization (HPLC-PCD-FLD), and HPLC-UV to analyze food samples. These combined technologies have high accuracy and recovery. However, the synthesis methods of carbon-based magnetic materials such as carbon nanotubes and graphene, incur high energy consumption and high cost, and involve complex processes, which limit their application. Therefore, a carbon-based magnetic adsorbent with low cost, high selectivity, and high extraction efficiency was developed by further exploring functional modification with biochar as a carbon base. This is a very promising direction to develop MSPE technology utilizing biochar-based magnetic materials for food sample pretreatment. This review provides a theoretical basis and technical support for the wide application of carbon-based magnetic materials in MSPE technology for food analysis.

各种农兽药残留、化学毒物、食品添加剂等可通过食品进入人体,严重威胁人体健康。因此,食品安全引起了社会各界的高度关注,食品中霉菌霉素等物质的分析已成为分析科学的重要研究课题^[1]^。对实际食品样品中微量/痕量有害物质进行测定和分析时,常由于目标分析物浓度低于仪器检出限等问题而无法获得分析结果。因而,面对目标分析物种类繁多、样品痕量(超痕量)、样品基质复杂等挑战,需引入适当的样品预处理技术,以便在仪器分析之前分离和富集目标分析物^[2]^。固相萃取是常见的食品样品预处理方法,是利用固体吸附剂吸附液体样品中的目标分析物,使其与样品基质和干扰物分离,再用洗脱液洗脱或加热解吸附,达到分离、净化、富集的效果^[3]^。然而,固相萃取与液液萃取等方法类似,存在选择性较差、受基质干扰影响大、样品需要量较大、萃取时间长、使用大量有害有机溶剂、操作烦琐耗时等问题。磁性固相萃取(MSPE)是将磁性吸附剂加至样品溶液中,吸附萃取目标分析物,待萃取完成后通过外加磁场将磁性吸附剂与样品溶液分离,在对吸附剂进行解吸后,即可进行定性定量分析^[4]^。

磁性固相萃取综合了磁性分离和传统固相萃取技术的优点,避免了装柱耗时的问题,可高效提取目标分析物,且无需离心或过滤即可实现目标分析物与干扰物间的快速分离^[5]^,具有操作简单、省时快速、无需离心过滤、环境友好等优点,在食品分析中显示出良好的潜力^[6]^。磁性吸附剂的吸附容量和选择性是影响MSPE萃取效率和选择性高低的关键,对所建立方法的分析准确度起着关键作用^[7]^。碳基磁性材料是利用碳基材料(碳纳米管、石墨烯、金属有机骨架衍生碳、活性炭等)和磁性材料通过共沉淀等方法制备的磁性复合材料。碳基磁性材料进行适当功能化修饰后,不仅兼具碳材料和磁性材料的优点,同时可体现出比表面积大、稳定性良好、成本低、环境友好、物化性能优异、孔隙率和吸附容量高等优点。它是一类新型功能性磁性材料,可以富集不同性质的有机、无机分析物,在环境分析、生物检测、污染治理等多个领域取得了较大进展。近年来,碳基磁性材料被开发为具有较好应用前景的MSPE磁性吸附剂^[8]^。查阅SCI和中文核心期刊上近4年50多篇国内外发表的相关文献后,获悉碳基类别包括碳纳米管、石墨烯、氧化石墨烯、还原性氧化石墨烯、金属有机骨架衍生碳、活性炭、生物炭、纳米金刚石等。该文以碳基类别为主线,综述了采用MSPE技术,以碳基磁性材料为吸附剂,对食品样品中酯类、真菌毒素、多环芳烃、抗生素、生物碱、酚类、维生素、抗菌药等物质进行萃取,进而建立MSPE与液相色谱法等联用技术进行分析的现状及存在的问题,并对其发展方向做出展望,为基于碳基磁性材料的MSPE技术在食品分析中的广泛应用提供理论依据和技术支撑。

## 1 碳纳米管基

碳纳米管具有孔隙率高、比表面积大、易于功能化等优点,是MSPE的理想吸附剂^[9]^。然而,磁性碳纳米管(MCNTs)依靠弱的*π-π*相互作用和疏水作用,对多种类型分析物的萃取性、敏感性和选择性较低^[9]^,若采用官能团、功能试剂或功能材料对磁性碳纳米管进行功能修饰,引入氢键、静电、配位作用和分子尺寸选择效应等因素,则可显著提高功能性磁性碳纳米管的萃取性能。

### 1.1 官能团

自2015年起,很多研究学者采用酸化改性法将羟基和羧基官能团修饰到多壁碳纳米管上,改善碳纳米管的活性吸附位点,增MCNTs对蜂蜜中6种菊酯类农药残留物^[10]^、酸奶饮料中6种邻苯二甲酸酯^[11]^、牛奶和奶粉样品中16种邻苯二甲酸酯^[12]^的吸附亲和力,相应建立起的分析方法准确度等较高(见表1)。氨基修饰的MCNTs萃取性能得到进一步提高,对牛奶中13个磺胺类药物应用MSPE-LC-HRMS联用方法分析,精密度较高(RSD为0.9%~6.2%)^[13]^。氮作为掺杂剂被掺入碳纳米管的石墨晶格中,在碳纳米管壁上形成一定数量的缺陷点,氮功能化磁性碳纳米管的化学活性和表面亲水性都得到了显著提高,对分析物的吸附亲和力也得到了有效提高,可达到对果汁中4种双酚类物质的高效预浓缩^[14]^。

**表 1:** 基于碳基磁性材料的磁固相萃取技术在食品分析中的应用

Carbon base	Carbon based magnetic composite material	Analyte	Samples	Method	LOD	Recovery/ %		RSD/ %	Ref.
CNTs	Fe_3_O_4_/AMWNTs	pyrethroid pesticides	honey	GC	0.07-0.20 μg/L	78.4	-94.8	3.8-8.1	[10]
	MWCNT-Fe_3_O_4_	phthalate acid esters	yogurt-based drinking	GC-MS	10.00-24.00 ng/L		-	0.3-7.4	[11]
	MWCNT-MNP	polycyclic aromatic hydrocarbons	milk, milk powder	GC-MS	0.040-0.075 μg/kg	86.1	-100.3	3.2-10.1	[12]
	Tol-MCNT	sulfonamides	milk	LC-HRMS	2.00-10.00 ng/L	86.6	-98.3	0.9-6.2	[13]
	M-*N*-CNTs	bisphenols	fruit juices	UHPLC-MS/MS	0.43-2.47 ng/L	90.6	-101.0	1.2-5.8	[14]
	PAMAM@Mag-CNTs	toxic alkaloids	meat, vegetable	UFLC-MS/MS	0.011-0.329 ng/g	83.4	-125.0	1.3-8.0	[15]
	PEG-MWCNTs-MNP	mycotoxins	real liquid milk	UHPLC-Q-Exactive HRMS	0.005-0.050 μg/kg	81.8	-106.4	2.1-8.5	[16]
	HCSs@Fe_3_O_4_- MWCNT-COOH	herbicides	wheat flour	HPLC-DAD	0.24-0.68 ng/g	88.8	-96.6	1.6-6.2	[17]
	Fe_3_O_4_/MWCNT@ND	vitamin B12	infant formula powder, cereal	HPLC-DAD	2.85 ng/mL	97.8	-103.1	4.3-6.1	[18]
	*N*-CNTCs	okadaic acid	mussel, oyster	HPLC-MS/MS	1.30 pg/mL	82.0	-107.0	1.8-2.5	[19]
	mMWCNT-ZrO_2_-C18	polycyclic aromatic hydrocarbons	edible oils	HPLC-DAD	0.06-0.55 ng/g	93.9	-113.3	3.9-6.0	[20]
G	Fe_3_O_4_@SiO_2_@G	preservative	vegetables	GC-MS	0.21-11.50 μg/kg	78.3	-116.7	1.4-11.9	[21]
	MG@SiO_2_-TMSPED	pesticides	tomato, grape	GC-μECD	0.23-0.30 μg/kg	82.0	-113.0	5.1-8.1	[22]
	mNi@N-GrT	tetracyclines	milk	HPLC	1.29-2.31 ng/mL	91.6	-109.7	2.2-5.7	[23]
	3D-G-Fe_3_O_4_	caffeine	food	GC-FID	0.10 μg/mL	93.1	-97.7	5.9-7.1	[24]
	3D-G-Fe_3_O_4_	organophosphorus pesticides	juice	GC-NPD	1.20-5.10 ng/L	86.6	-107.5	2.5-7.4	[25]
	3D-G-Fe_3_O_4_	chlorophenols	honey	HPLC-UV	1.00-1.50 ng/g	93.2	-98.9	4.1-4.6	[26]
GO	Fe_3_O_4_@GO	sulfonamides	milk	HPLC-MS/MS	0.02-0.13 μg/L	73.4	-97.4	1.0-8.2	[27]
	Fe_3_O_4_@SiO_2_-GO/ MIL-101(Cr)	triazine herbicides	rice	HPLC	0.01-0.08 μg/kg	83.9	-103.5	0.5-8.7	[28]
	MGO	phthalate esters	bottled water	HPLC	0.004-0.013 mg/L	65.0	-126.0	0.6-6.0	[29]
	Fe_3_O_4_/GO	melamine	dairy products	HPLC	0.03 μg/L	97.2	-103.1	1.1-5.0	[30]
	Fe_3_O_4_/rGO	aflatoxins	vegetable oils	HPLC-PCD-FLD	0.01-0.02 μg/kg	80.4	-106.0	1.3-10.5	[31]
	rGO/ZnFe_2_O_4_	estrogens	fish	HPLC-DAD	0.01-0.02 ng/mL	73.5	-104.1	1.7-8.3	[32]
MOFs	MNPCs	organophosphorus pesticides	fruit	GC-FPD	0.018-0.045 μg/L	84.0	-116.0	3.5-9.7	[33]
AC	MBAC	phenylurea herbicides	juice	HPLC-UV	0.10-0.80 ng/mL	86.1	-104.1	4.0-6.8	[34]
BC	C/Fe_3_O_4_ NCs	triazole fungicides	fruit	GC-MS	0.12-0.55 μg/kg	82.1	-109.9	2.1-8.4	[35]
GC	YS-Fe_3_O_4_@GC	sulfonamides	milk, meat	HPLC	0.11-0.25 μg/L	77.2	-118.0	1.4-9.2	[36]

CNTs: carbon nanotubes; AMWNTs: acid multi-walled carbon nanotubes; MWCNT-MNP: hybrids of magnetite (Fe_3_O_4_) with multiwalled carbon nanotube (MWCNT); Tol-MCNT: *p*-tolyl-functionalized magnetic carbon nanotubes; M-*N*-CNTs: magnetic nitrogen doped carbon nanotubes; UHPLC: ultra high performance liquid chromatography; PAMAM@Mag-CNTs: polyamidoamine-functionalized magnetic carbon nanotubes; UFLC: ultra-fast liquid chromatography; PEG-MWCNTs-MNP: pegylated multi-walled carbon nanotubes magnetic nanoparticles; HCSs: hollow carbon nanospheres; ND: nanodiamond; *N*-CNTCs: magnetic nitrogen doped carbon nanotube cages; mMWCNT-ZrO_2_-C18: magnetic multiwalled carbon nanotubeoctadecylphosphonic acid modified zirconia; G: graphene; MG@SiO_2_-TMSPED: magnetic graphene based hybrid silica-*N*-[3-(trimethoxysilyl)propyl]ethylenediamine; μECD: micro-electron capture detection; mNi@N-GrT: ultrathin magnetic nitrogen doped graphene tube; 3D-G-Fe_3_O_4_: magnetic three dimensional-graphene nanocomposite; NPD: nitrogen phosphorous detection; GO: graphene oxide; Fe_3_O_4_@SiO_2_-GO/MIL-101(Cr): metal-organic framework functionalized magnetic graphene oxide/mesoporous silica composites; MGO: magnetic graphene oxides; rGO: reduced graphene oxide; PCD: post-column photochemical derivatization; MOFs: metal-organic framework; MNPCs: magnetic nanoporous carbons; FPD: specific flame photometricdetector; AC: activated carbon; MBAC: magnetic biomass activated carbon; BC: biochar; C/Fe_3_O_4_ NCs: carbon-based Fe_3_O_4_ nanocomposites; YS-Fe_3_O_4_@GC: yolk-shell Fe_3_O_4_@graphitic carbon.

### 1.2 功能试剂

采用具有特异识别能力和高吸附选择性的功能性试剂(金属和金属化合物、树枝状大分子、有机聚合物)对碳纳米管进行改性,通过增加*π-π*堆积、氢键、偶极-偶极和离子偶极、疏水相互作用等,可以显著提高磁性碳纳米管对目标分析物的吸附性和选择性,促进碳纳米管在MSPE技术中更好的应用。Moazzen等^[37,38]^采用化学共沉淀法将银(Ag)固定在碳纳米管表面,可作为催化剂和表面增强剂,制备的磁性复合材料磁性多壁碳纳米管/Fe_3_O_4_/Ag(MWCNT/Fe_3_O_4_/Ag),可作为分离和测定不同碳酸饮料样品中6种邻苯二甲酸酯的有效吸附剂。Razmkhah等^[39]^利用超声作用,将合成的SrTiO_3_沉积在经过酸化改性后磁化的磁性碳纳米管表面上,合成了一种新型磁性碳纳米管基锶钛(Fe/CNT-SrTiO_3_)吸附剂,从牛奶中提取了两种甾体激素(17*β*-雌二醇和孕酮)和一种雌二醇的合成衍生物(炔雌二醇)。此外,通过磁性碳纳米管表面的羧基与聚酰胺-胺(PAMAM)树枝状大分子的外围氨基直接反应,合成聚酰胺-胺功能化磁性碳纳米管PAMAM@Mag CNTs,可增强吸附剂与中草药食谱中15种生物碱在萃取过程中的相互作用,具有较高的萃取效率和回收率^[15]^。Zhao等^[16]^制备的聚乙二醇化多壁碳纳米管磁性纳米粒(PEG-MWCNTs-MNP)在奶制品中的稳定性和均匀性显著提高,以其作为吸附剂,对液态奶中13种真菌毒素应用MSPE结合超高效液相色谱-精确高分辨质谱联用方法分析,具有较高灵敏度和特异性(见表1)。

### 1.3 功能材料

用功能材料(碳材料、金属有机骨架、功能复合材料等)对磁性碳纳米管进行改性后,磁性碳纳米管具有更高的萃取能力和选择性,从而在磁固相萃取预处理食品样品应用中具有广阔的前景。因空心碳纳米球(HCSs)增强的磁性羧基碳纳米管(HCSs@Fe_3_O_4_ MWCNTs-COOH)的羧基数量和结构间距较MWCNTs-COOH有所增加,使得该吸附剂的亲水性、分散性、萃取性和选择性都有所提高,借助该萃取剂萃取小麦面粉样品中双酚、二氯苯和甲基双氯酚,配合HPLC-DAD进行测定,精密度和回收率高,LOD小于0.68 ng/g^[17]^。纳米材料因其体积小、比表面积大、吸附容量高,而受到了广泛的关注。纳米金刚石(NDs)具有优异的力学性能和高比表面积,这使其成为潜在的优越吸附剂。用NDs修饰MWCNTs制备的一种磁芯-壳MWCNT@ND磁性萃取材料稳定性较好,能有效萃取婴儿配方奶粉和早餐谷类食品中的微量维生素B12^[18]^。以含钴(Ⅱ)金属有机骨架为原料,可直接碳化制备磁性氮掺杂碳纳米管*N*-CNTCs。碳化过程中,MOFs中的钴离子被转化为*N*-CNTCs的磁性纳米粒子。同时,大量来自有机配体的氮被掺杂到碳骨架中。这种独特的结构赋予了*N*-CNTCs优异的化学稳定性,高亲和力和良好的分散性,可用于水产样品(贻贝和牡蛎)中大田软海绵酸的预浓缩^[19]^。氧化锆(ZrO_2_)具有优异的机械和热稳定性,良好的萃取效率,高韧性和生物相容性。采用简单的溶剂热还原法制备的磁性多壁碳纳米管十八烷基膦酸改性氧化锆mMWCNT-ZrO_2_-C18吸附剂,对在食用油样品中多环芳烃的预富集中体现出更高的回收率和更好的吸附性能,具有重要的应用前景^[20]^。

## 2 石墨烯基

### 2.1 石墨烯

石墨烯与碳纳米管一样,具有高比表面积,良好的稳定性和优异的物理化学性能,被认为是样品制备中提取和富集碳基环结构的理想材料^[40]^。为了进一步提高石墨烯基磁性材料的灵敏度、稳定性、吸附容量和萃取回收率,需要对其进一步改性^[41]^。研究结果表明,石墨烯接枝二氧化硅涂层磁性材料(Fe_3_O_4_@SiO_2_@G)、磁性石墨烯基杂化二氧化硅-*N*-[3-(三甲氧基硅基)丙基]乙二胺纳米复合材料(MG@SiO_2_-TMSPED)、超薄磁性氮掺杂石墨烯管(mNi@*N*-GrT)3种改性石墨烯基磁性材料分别能快速、简便、高效萃取蔬菜样品中16种防腐剂^[21]^、番茄和葡萄中3种农药^[22]^、奶制品中3种四环素类化合物^[23]^。三维石墨烯具有快速离子/电子传输、高比表面积、多孔和中空结构和强机械性能的理想特性^[42]^,能很好解决石墨烯片由于强堆积、疏水相互作用和范德华力的作用引起的团聚现象,是一种有前途的高效吸附剂。利用自组装法可合成三维石墨烯-Fe_3_O_4_(3D-G-Fe_3_O_4_)纳米颗粒,将其引入MSPE工艺,可对食品中咖啡因^[24]^、5种果汁(苹果、橘子、葡萄、酸樱桃和杏果汁)中8种有机磷农药^[25]^、蜂蜜样品中的氯酚^[26]^进行高效提取和准确测定(见表1)。

### 2.2 氧化石墨烯

GO由石墨烯在强氧化剂作用下剥落氧化而成。石墨烯比表面积大,且表面有许多环氧基、羟基、羧基,因此GO在实际应用中具有较高的吸附容量。通过在GO结构中引入磁性材料,可以实现更高吸附容量,便捷分离环境水样中的7种内分泌干扰物^[43]^。Fe_3_O_4_/GO磁性氧化石墨烯纳米复合材料对牛奶中15种磺胺类药物^[27]^、稻谷样品中7种三嗪类除草剂^[28]^、瓶装水中5种邻苯二甲酸酯^[29]^和乳制品中三聚氰胺^[30]^有较好的萃取效果,配合HPLC方法测定,检出限低,灵敏度高,线性范围宽(见表1)。

### 2.3 还原型氧化石墨烯

rGO含有大量含氧原子的极性基团,包括羟基、环氧基和羧基,它们通过配位键、阳离子相互作用、静电相互作用或氢相互作用,对有机污染物的氧和氮官能团表现出最佳的吸附能力。为了防止rGO的聚集,Yu等^[31]^采用简单的一锅溶剂热法合成了磁性石墨烯纳米复合材料(Fe_3_O_4_/rGO),将其作为MSPE吸附剂,与高效液相色谱-在线柱后光化学衍生-荧光检测法联用,可简便、快速、高效地测定食用植物油中黄曲霉毒素的含量。为解决磁性复合材料中Fe_3_O_4_纳米粒子在酸性溶液中不稳定的问题,Li等^[32]^将具有独特的物理化学性质、良好的稳定性、无毒性、高比表面积和优异磁性的锌铁氧体(ZnFe_2_O_4_)代替Fe_3_O_4_,采用一步水热法合成了还原氧化石墨烯/ZnFe_2_O_4_(rGO/ZnFe_2_O_4_)磁性纳米复合材料,并将其作为MSPE的吸附剂,用于鱼类样品中4种雌激素(17*β*-雌二醇、17*α*-雌二醇、雌酮、己雌酚)的提取和富集,所建立的分析方法达到较好的准确度(见表1)。该方法对环境和生物样品等复杂基质中超痕量化合物的提取和富集具有潜在的应用前景。

## 3 金属有机骨架衍生碳基

MOFs是一类由金属团簇和有机连接体共价连接而成的杂化多孔材料,具有比表面积大、结构有序、孔隙率高等优异性能,因此被广泛作为吸附剂用于样品前处理中^[44]^。同时,MOFs碳含量高,是炭化过程中良好的碳前驱体。在惰性气体中直接煅烧MOFs,制备出磁性MOFs衍生纳米多孔碳/衍生碳的方法简单,适合工业化生产^[45]^。Li等^[33]^采用氧化锌纳米粒、Co(OH)_2_和2-甲基咪唑混合液炭化法合成了金属有机骨架衍生磁性纳米多孔炭Zn/Co-MOFs磁性纳米多孔炭。碳化过程中Co纳米颗粒的形成而具有很强的磁性,且不需要消耗有机溶剂。该复合材料具有高比表面积,大孔体积,可调有序孔结构,以及良好的化学稳定性,以其作为MSPE吸附剂,并结合GC-FPD,建立了水果样品中5种痕量OPPs(对甲硫磷、二嗪酮、乙硫磷、马拉硫磷、芬硫磷)的定量分析方法(见表1)。该方法简便、快速,选择性强,灵敏度高,可有效降低试剂用量和成本,用于各种水果样品中痕量有机磷的分析。

## 4 活性炭基

AC具有比表面积大、表面官能团范围广、内部微孔等优点,是一种用于混合物分离纯化的有效吸附剂,在许多领域得到了广泛应用。磁性活性炭是由非磁性活性炭和磁棒组成的复合材料^[46]^。Filippou等^[47]^采用浸渍法成功将木质活性炭制备成磁性活性炭(Bmi),该吸附剂中含有10%磁铁矿,在整个表面上分布有缺陷和空穴的海绵状结构,与双酚A存在氢键作用和苯环的电子相互作用,对牛乳和人乳中双酚A的最大吸附容量可达353.39 mg/g。Wang等^[34]^以农业废弃花生壳为原料,经磷酸活化、400 ℃炭化制备了生物活性炭,加入磁性组分,进而制备的新颖、环境友好、低成本、环保、高效的磁性生物活性炭,可从瓶装玫瑰汁样品中简单、快速地萃取4种苯脲类除草剂(绿麦隆、异丙隆、单宁隆和布图隆)。

## 5 其他碳材料基

各种天然生物质如玉米秸秆^[48]^、花生壳^[49]^等被广泛应用于制备磁性生物炭(MBC)。区别于磁性生物活性炭,MBC仅经过低温碳化处理(低于400 ℃),即具有一定的吸附性能。柚子皮占新鲜水果的44%~54%,若直接丢弃,会导致环境问题。然而,柚子皮中含有丰富的植物纤维和羟基、羧基、酰胺基等官能团,使其成为一种有前途的吸附剂^[50]^。Ren等^[35]^以废弃的海绵状柚皮为碳前驱体,采用一步水热法(180 ℃下进行水热碳化15 h)合成了碳基Fe_3_O_4_纳米复合材料(C/Fe_3_O_4_ NCs),其近似为球形(直径约为50 nm),在室温下饱和磁化强度为45.9 emu/g,具有超顺磁性,水热过程中碳化使得表面含有丰富的含氧基团(O-H和C=O等),易于在水中分散,并在外加磁场作用下分离,可从苹果、梨、橘子和香蕉样品中高效萃取11种三唑类杀菌剂(见图1)。

**图 1:**
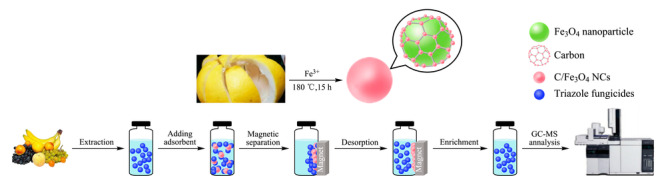
C/Fe_3_O_4_ NCs的合成工艺及MSPE分析水果中三唑类杀菌剂的步骤说明图^[35]^

Yilmaz等^[51]^对NDs进行了磁性羧基改性,继而将纳米氧化铁颗粒包覆在其表面形成核壳结构的磁性羧化纳米金刚石,该吸附剂的磁性、分散性和水溶液稳定性较好,且表面含有多个结合位点,对食品样品中痕量的农药二甲基二硫代氨基甲酸锌(Ziram)的萃取速度快(小于10 min),操作简便,可以在不损失任何磁性和吸附性能的情况下使用15次,检出限低。亚微盒中合适的内腔结构、石墨碳壳结构(包含共轭*π*结构)和较大的比表面积对提高分析物的传质性能具有重要作用。与核壳结构相比,Liu等^[36]^制备了一个内腔可调的蛋黄壳Fe_3_O_4_@石墨碳(YS-Fe_3_O_4_@GC)亚微盒,对牛奶和肉类样品中4种痕量磺胺类药物(SAs)具有优越的富集性能(见表1),为蛋黄壳结构的可调制备提供了一种新思路。

## 6 总结与展望

综上所述,碳基磁性材料具有良好的预富集能力,基于碳基磁性材料的MSPE技术有巨大的应用潜力。然而基于碳基磁性材料的MSPE在食品分析中的应用尚处于起步阶段,碳基磁性吸附剂因氢键和*π-π*相互作用,对多芳环结构、多氢键受体和大分子极性比表面积的分析物敏感,预处理研究的目标分析物质主要集中在食品样品中的酯类、真菌毒素、多环芳烃、抗生素、生物碱、酚类、维生素、抗菌药等;碳基磁性材料中的碳基如碳纳米管和石墨烯的合成方法能耗高、成本高、工艺复杂等缺陷限制了其应用。因此,以来源广泛廉价的生物炭作为碳基,进一步探索功能化修饰,开发低成本、选择性高、萃取效率高的碳基磁性吸附剂,将拓宽基于碳基磁性材料的MSPE技术在食品样品分析中的应用。
